# FBXO5 regulates RPL23A to promote MDM2-mediated p53 degradation and facilitate malignant progression of breast cancer

**DOI:** 10.1186/s13058-026-02335-3

**Published:** 2026-06-25

**Authors:** Jianwen Wang, Longdi Yao, Yuming Chen, Qiang Zhu, Wenjing Xu, An Xu, Xiang Li, Zhi Chen, Min Cheng, Qi Leng, Chunlian Li, Xintao Deng, Deyuan Fu

**Affiliations:** 1 Department of Thyroid and Breast Surgery, Xinghua People’s Hospital Affiliated to Yangzhou University, Xinghua, Jiangsu Province China; 2 Department of Thyroid and Breast Surgery, Changxing Hospital of Traditional Chinese Medicine, Huzhou, China; 3 Department of Urology, Xinghua People’s Hospital Affiliated to Yangzhou University, Xinghua, Jiangsu Province China; 4 Department of Nephrology, Xinghua People’s Hospital Affiliated to Yangzhou University, Xinghua, Jiangsu Province China; 5 Department of Medical Oncology, Xinghua People’s Hospital Affiliated to Yangzhou University, Xinghua, Jiangsu Province China; 6 Department of General Surgery, Northern Jiangsu People’s Hospital Affiliated to Yangzhou University, Yangzhou, Jiangsu Province China; 7 Department of Neurology, Xinghua People’s Hospital Affiliated to Yangzhou University, Xinghua, Jiangsu Province China; 8 Department of Clinical Laboratory Medicine, Xinghua People’s Hospital Affiliated to Yangzhou University, Xinghua, Jiangsu Province China; 9 Department of Pathology, Xinghua People’s Hospital Affiliated to Yangzhou University, Xinghua, Jiangsu Province China; 10 Department of Cardiology, Xinghua People’s Hospital Affiliated to Yangzhou University, Xinghua, Jiangsu Province China

**Keywords:** Breast cancer, Ubiquitination, FBXO5, RPL23A, MDM2, p53

## Abstract

**Background:**

Breast cancer (BC) is one of the most common malignant tumors in women worldwide, with metastasis and recurrence constituting major therapeutic challenges. F-Box Protein 5(FBXO5), a core component of the E3 ubiquitin ligase complex, is overexpressed in multiple cancers, but its specific role and underlying mechanism in BC remain unclear and require further investigation.

**Methods:**

We screened E3 ubiquitin ligase-related cancer dependency genes by integrating data from the DepMap and UniProt databases, and further analyzed transcriptomic data from TCGA and GEO to identify the candidate gene FBXO5. We then validated its expression at the mRNA and protein levels in BC tissues from two independent centers and in cell lines, employing qRT-PCR, Western blot, and immunohistochemistry.The effects of FBXO5 on the proliferation, migration, and invasion abilities of BC cells were evaluated in vitro and in vivo through colony formation, CCK8, Transwell assays, wound healing assays, IHC, and subcutaneous tumor formation in nude mice. The interactions between FBXO5 and proteins, as well as its main functions and pathways, were investigated using Co-IP, mass spectrometry, immunofluorescence confocal microscopy, molecular docking and bioinformatics analysis, with results validated by rescue experiments.

**Results:**

The mRNA and protein expression levels of FBXO5 were significantly upregulated in BC tissues from two centers, high expression of FBXO5 was significantly correlated with adverse clinicopathological features, including larger tumor size, positive nodal status, and elevated Ki-67, and was associated with poor overall survival (OS) and disease-free survival (DFS).In vitro and in vivo experiments confirmed that FBXO5 significantly affected the proliferation, invasion, and migration abilities of BC cells. Mechanistically, Co-IP, mass spectrometry, molecular docking and immunofluorescence confocal microscopy experiments confirmed that FBXO5 directly interacts with ribosomal protein L23a (RPL23A) and promotes its polyubiquitination at the K48 chain, thereby regulating its degradation. Further experiments showed that the ubiquitination-mediated degradation of RPL23A led to a decrease in the stability of Tumor Protein p53(p53) and facilitated its degradation by Proto-Oncogene MDM2 (MDM2).

**Conclusion:**

Our study establishes the existence of a novel FBXO5/RPL23A/MDM2/p53 oncogenic axis in BC. These findings thereby nominate FBXO5 as a promising therapeutic target for BC intervention.

**Supplementary Information:**

The online version contains supplementary material available at https://doi.org/10.1186/s13058-026-02335-3.

## Background

Breast cancer(BC) is the most common malignant tumor among women worldwide and remains one of the leading causes of cancer-related deaths in women [1]. In recent years, the incidence of BC has continued to rise, presenting a significant challenge for patient treatment and management. Although modern medicine has expanded treatment strategies for BC beyond traditional surgery to include chemotherapy, radiotherapy, endocrine therapy, targeted therapy, and immunotherapy, which have significantly improved overall survival rates(OS), the infiltration and distant metastasis of BC remain the primary causes of patient mortality [2]. Improving the precision and efficacy of treatments, as well as reducing the risks of recurrence and metastasis, remains a major challenge in the clinical management of BC. With advances in genomics, transcriptomics, and proteomics, particularly the rapid development of single-cell sequencing technologies, our understanding of the pathogenesis of BC has deepened [3–4]. However, the molecular mechanisms underlying the occurrence, progression, and metastasis of BC are highly complex and are regulated by a network of genes and signaling pathways that have not yet been fully elucidated. Therefore, there is an urgent need for in-depth studies on the molecular mechanisms of BC to discover novel biomarkers and therapeutic targets, ultimately providing more precise and effective diagnostic and therapeutic strategies for BC patients.

The occurrence and progression of BC is a complex biological process driven by multiple genes, factors, and stages, relying on the intricate interactions and precise regulation between genes, transcripts, and proteins [5]. In recent years, functional genomic screening technologies based on the clustered regularly interspaced short palindromic repeat-associated protein 9 (CRISPR/Cas9) have emerged as powerful tools for identifying critical tumor targets, owing to their efficiency, reliability, and objectivity. This technology allows for the identification of potential genes that play key roles in tumor cell proliferation and survival, providing new therapeutic targets for cancer treatment [6–8]. The Cancer Dependency Map (DepMap) integrates CRISPR-Cas9 gene editing and related technologies to perform genome-wide gene knockout across thousands of tumor cell lines, revealing the roles of various genes in tumor cells [9]. Overexpression of genes that promote tumor development and progression may lead to tumor recurrence, metastasis, and resistance. In our study, we define these genes as Cancer Dependency Genes (CDGs). The “Cancer Dependency Map” constructed by DepMap provides a systematic analytical framework for the dependency relationships between specific genes and different cancer types, which aids in the identification of dependency genes closely associated with tumor proliferation and survival [10].

The Ubiquitin-Proteasome System (UPS) is a crucial protein degradation mechanism within cells, regulating post-translational modifications of proteins. During cancer initiation and progression, the UPS is involved in key biological processes such as cell cycle progression, cell proliferation, and apoptosis [11]. Among its components, E3 ubiquitin ligases play a central role in the UPS due to their ability to specifically recognize target proteins, making them a focal point in cancer research [12].

F-Box Protein 5 (FBXO5), a member of the F-box family, is a subunit of the E3 ubiquitin ligase complex SKP1-Cullin1-Fbox (SCF). It plays a crucial role in various cellular processes through the ubiquitination and degradation of multiple proteins [13]. FBXO5 was initially identified as an endogenous inhibitor of the anaphase-promoting complex/cyclosome (APC/C) in a yeast two-hybrid screen and is a key regulator of the cell cycle [14–16]. Recent studies have shown that FBXO5 is upregulated in various cancers and is positively correlated with poor prognosis, including liver cancer, lung cancer, esophageal cancer, BC, and ovarian cancer [17–21]. However, research on FBXO5 remains limited, and its functional significance in cancer development and progression is not yet fully understood. In particular, the specific oncogenic mechanisms of FBXO5 in BC are unclear. Elucidating its potential oncogenic mechanisms may provide insights into the diagnosis and treatment of BC.

This study investigated the expression of FBXO5 in BC tissues and its impact on patient prognosis, with a focus on elucidating the regulatory mechanisms of FBXO5 in BC cell proliferation. The findings provide new strategies for the selection of targeted therapies for BC.

## Materials and methods

### Bioinformatics analysis

The CERES algorithm available on the DepMap website was employed to generate dependency scores for 18,444 genes across 1,150 cell lines [22]. A negative score indicates that knockdown of the corresponding gene suppresses cell survival and proliferation. From 51 BC cell lines, 573 candidate genes with CERES scores less than − 1 in at least 80% of the cell lines were identified. A total of 1,244 human E3 ubiquitin ligases were retrieved from the UniProt database. The intersection between these two gene sets was visualized using a Venn diagram. The complete lists of DepMap candidate genes, UniProt-derived E3 ubiquitin ligases, and their intersection (CDGs) are provided in Supporting Information S1.

Transcriptomic data from 1,081 BC samples and 99 adjacent normal tissues were obtained from the Cancer Genome Atlas (TCGA) database via UCSC Xena. Differential expression analysis of E3 ubiquitin ligase-associated CDGs was performed using the “DESeq2” package. Additionally, transcriptomic data from 109 BC samples and 24 adjacent normal tissues were retrieved from the Gene Expression Omnibus (GEO) database (GSE162228 dataset), and differential expression analysis of the same set of E3 ubiquitin ligase-related CDGs was conducted using the “limma” package. The intersection of differentially expressed genes between the two datasets was visualized using a Venn diagram. The resulting DEGs from both datasets and their intersection are provided in Supporting Information S2. The expression differences of FBXO5 between various cancer tissues and normal tissues were assessed using the GEPIA database. Prognostic analysis of FBXO5 was performed using the Kaplan-Meier Plotter database.

FBXO5 genomic mutation and copy number variation (CNV) were analyzed using the TCGA Breast Invasive Carcinoma (PanCancer Atlas) dataset in cBioPortal. The correlation between FBXO5 methylation level and mRNA expression was assessed through the cBioPortal “Plots” module, and both Spearman and Pearson correlation analyses were performed to determine correlation coefficients and *P* values. UALCAN database was used to compare FBXO5 promoter methylation levels between breast cancer tissues and normal tissues in the TCGA dataset.

### Specimen collection

This study included two independent cohorts of BC patients. The first cohort comprised 180 patients who underwent surgical resection at Northern Jiangsu People’s Hospital Affiliated to Yangzhou University, between January 2016 and December 2020. Tumor tissues and matched adjacent non-tumor tissues were collected, and tissue microarrays were subsequently constructed by Servicebio Biotechnology Co., Ltd. The second cohort included 120 patients who underwent surgery at Xinghua People’s Hospital Affiliated to Yangzhou University, between January 2017 and December 2020. Tumor and matched adjacent non-tumor tissues were collected and prepared as conventional paraffin-embedded sections. In addition, between January 2025 and June 2025, eight pairs of fresh-frozen BC tissues and matched adjacent tissues were obtained from each of the two hospitals.The use of all clinical specimens was reviewed and approved by the Ethics Committee of Northern Jiangsu People’s Hospital Affiliated to Yangzhou University (Approval No. 2024ky381), and the Ethics Committee of Xinghua People’s Hospital Affiliated to Yangzhou University (Approval No. XRYLL-KY-2025002). Written informed consent was obtained from all participating patients.

### Cell lines and lentivirus infection

The normal mammary epithelial cell line MCF10A, the BC cell lines MCF7, SUM159PT, CAL51, SKBR3, and MDA-MB-231, as well as the HEK293T cell line used for lentiviral packaging, were all purchased from Zhongqiao Xinzhou Biotechnology Co., Ltd. (Shanghai, China). BC cell lines and HEK293T cells were cultured in high-glucose DMEM medium (Servicebio, China) supplemented with 10% fetal bovine serum (FBS; Servicebio, China) and 100 U/mL penicillin/streptomycin (Beyotime, China). MCF10A cells were cultured in a specific medium (Procell, CM-0525). All cells were maintained in a humidified incubator at 37 °C with 5% CO₂ (Thermo, USA).Lentiviral plasmid vectors for FBXO5 overexpression and knockdown, along with their respective controls, were obtained from Shandong Weizhen Biotechnology Co., Ltd. Plasmids for RPL23A overexpression and its control, as well as ubiquitin (HA-Ub) overexpression plasmids, were purchased from Nanjing Corewise Biotechnology Co., Ltd. Plasmid transfections were performed using Lipofectamine 3000 (Invitrogen, USA) and Opti-MEM (Gibco, USA) according to the manufacturer’s instructions.

### Antibodies and reagents

This study utilized various antibodies and chemical reagents. The primary antibodies included: FBXO5 (BD-PN6322, Biodragon, used for WB; CY8259, Abways, used for IHC/IF/IP); RPL23A (16386-1-AP, Proteintech, used for WB/IHC/IP; ab223089, Abcam, used for IF); β-actin (66009-1-Ig, Proteintech, used for WB); p53 (10442-1-AP, Proteintech, used for WB/IP); MDM2 (AF0208, Affinity, used for WB/IP); Flag-tag antibody (66008-4-Ig, Proteintech); HA-tag antibody (51064-2-AP, Proteintech); Secondary antibodies for Western blot were HRP-conjugated goat anti-rabbit IgG (SA00001-2, Proteintech) and goat anti-mouse IgG (SA00003-1, Proteintech); Fluorescent secondary antibodies included mouse-derived secondary antibody (SA00003-2, Proteintech) and rabbit-derived secondary antibody (SA00013-2, Proteintech). The chemical reagents included: Bafilomycin A1 (Beyotime, China); CHX (Aladdin, China); MG132 (Beyotime, China); Nutlin-3 (Beyotime, China); and DAPI (Solarbio, China).

### Immunohistochemistry (IHC)

Formalin-fixed, paraffin-embedded tissue specimens were sectioned at a thickness of 4 μm. After deparaffinization in xylene and rehydration through a graded ethanol series, antigen retrieval was performed using EDTA(Beyotime, China). Endogenous peroxidase activity was blocked with H₂O₂, followed by blocking with 5% BSA (Beyotime, China). Sections were incubated with primary antibodies overnight at 4 °C, washed with PBST, and subsequently incubated with secondary antibodies at room temperature for 2 h. Immunoreactivity was visualized with DAB (Beyotime, China), followed by dehydration through graded ethanol, clearing in xylene, and mounting with neutral balsam.IHC scoring was independently performed by two pathologists (associate chief physicians or above) under double-blind conditions, based on both staining intensity and the proportion of positive cells. Staining intensity was graded as: negative = 0, weak = 1, moderate = 2, and strong = 3; the percentage of positive cells was scored as: <1% = 0, 1–25% = 1, 26–50% = 2, 51–75% = 3, and > 75% = 4. The final IHC score was calculated as the product of the two parameters, with scores ≤ 6 defined as low expression and > 6 defined as high expression.

### Immunofluorescent staining

Cells in the logarithmic growth phase were seeded on sterile coverslips placed in 24-well plates and cultured for 24 h. The cells were then fixed with 4% paraformaldehyde for 10 min and permeabilized with 0.1% Triton X-100 (Beyotime, China) for 5 min. After blocking with 5% BSA (Beyotime, China) at room temperature for 30 min, the cells were incubated with the primary antibodies overnight at 4 °C in a humidified chamber. Following three washes with PBS, the cells were incubated with the appropriate fluorescent secondary antibodies at room temperature for 1 h in the dark and then washed three times with PBS. The samples were mounted with an anti-fade mounting medium containing DAPI (Beyotime, China). Images were acquired using a Nikon laser scanning confocal microscope (Eclipse Ti-Al).

### Colony formation, CCK-8, migration, invasion, and wound healing assays

Cells were seeded into six-well plates and cultured for 14 days, followed by fixation with 4% paraformaldehyde for 30 min and staining with 0.1% crystal violet for 30 min. After air-drying at room temperature, colonies were photographed and quantified using ImageJ software to evaluate clonogenic capacity. For the wound healing assay, cells were grown in six-well plates to > 90% confluence, and a linear scratch was made across the monolayer using a sterile 200 µL pipette tip. After washing with PBS, the cells were cultured in serum-free medium, and images were captured at 0 h and 48 h under an inverted microscope to assess cell migration. Transwell migration and invasion assays were performed using chambers with 8 μm pore membranes. Cells pretreated with serum-free medium for 12 h were seeded into the upper chamber, while 700 µL of complete medium containing 20% FBS was added to the lower chamber. After incubation for 24 h at 37 °C, non-migrated cells on the upper surface were removed, and the migrated cells on the lower surface were fixed, stained with crystal violet, and counted. For invasion assays, the upper chamber was precoated with 60 µL of Matrix-Gel™ (Beyotime, China) and allowed to solidify at 4 °C overnight prior to seeding. For the CCK-8 assay, cells were seeded into 96-well plates, and CCK-8 reagent (Servicebio, China) was added at 0, 24, 48, and 72 h. Following incubation at 37 °C for 2 h, absorbance was measured at 450 nm using a microplate reader (Thermo, USA) to assess cell proliferation.

### Western blot assay

Cells or tissue samples were lysed in RIPA buffer (Solarbio, China) containing PMSF(Beyotime, China) and incubated on ice for 30 min with intermittent vortexing. The lysates were centrifuged at 12,000 × g for 15 min at 4 °C, and the supernatants were collected. Total protein concentrations were determined using a BCA protein assay kit (Servicebio, China). Protein samples were mixed with SDS-PAGE loading buffer (Beyotime, China), denatured at 95 °C for 5 min, separated by SDS-PAGE, and electrotransferred onto PVDF membranes (Merck Millipore, USA). The membranes were blocked with 5% non-fat milk at room temperature for 2 h, incubated with primary antibodies overnight at 4 °C, and then with secondary antibodies at room temperature for 1 h. After washing with TBST (0.1% Tween-20; Solarbio, China), the membranes were developed using an ECL(Beyotime, China) and visualized with the ChemiDoc XRS+ imaging system (Bio-Rad, USA). Band intensities were quantified using ImageJ software.

### RNA extraction and qRT‑PCR experiments

Total RNA was extracted from tissues and cells using TRIzol reagent (Invitrogen, USA), and RNA concentration and purity were measured with a NanoDrop spectrophotometer (Thermo, USA). Samples with an A260/A280 ratio between 1.8 and 2.0 were used for further analysis. cDNA was synthesized from RNA using a reverse transcription kit (Servicebio, China). Quantitative real-time PCR (qRT-PCR) was then performed with the SYBR Green qPCR Master Mix (Servicebio, China) on a real-time PCR detection system.The Forward primer for FBXO5(5-CCTCAGTGAACATGAAGAAGGTAGC-3), Reverse primer(5’-CTTTGTATTTGTAGCAGGCAGGATTG-3’), Forward primer.

for RPL23A (5’-TGCTATCATCAAATTCCCAC-3’),Reverse primer (5’- ACATACGCCTTCTTCTCTCC-3’). The Forward primer for GAPDH (5’-ACAGAGCCTCGCCTTTGCCGAT-3’), Reverse primer(5’-GGCCTCGTCGCCCAC ATAGGA-3’).

### Co-immunoprecipitation (Co-IP), mass spectrometry, and ubiquitination assay

Cells were first washed with PBS and subsequently lysed in buffer supplemented with 1 mM PMSF at 4 °C for 15 min. The lysates were collected into 1.5 mL microcentrifuge tubes, subjected to ultrasonication for 5 s, and centrifuged at 14,000 × g for 10 min at 4 °C. The supernatant was then incubated with 5 µg of primary antibody at 4 °C overnight, followed by the addition of Protein A + G agarose beads (Beyotime, China) and further incubation for 3 h. After three washes with PBS, the precipitated immune complexes were resuspended in 1× SDS loading buffer and boiled for 5 min. The resulting protein samples were subjected to Western blot analysis. Additionally, the co-immunoprecipitation products were submitted to Shanghai Zhongke Xin Shengming Biotechnology Co., Ltd. for mass spectrometry analysis.For the ubiquitination assay, the cells were treated with 20 µM MG132 for 8 h, and the protein–antibody complexes were extracted by Co-IP. The ubiquitination level of the target protein was subsequently examined by Western blot analysis.

### Mouse xenograft model

Female nude mice (4 weeks old, 15–21 g) were obtained from the Translational Medical Center at Yangzhou University. A total of 25 mice were randomly divided into five groups (*n* = 5 per group): control, OE-FBXO5, Sh-FBXO5, OE-FBXO5 + OE-RPL23A, and OE-FBXO5 + Nutlin-3 (Beyotime, China). SUM159PT stable cell lines with different transfection types were subcutaneously injected into the inguinal region of the mice. Tumor volumes were measured every 5 days after inoculation using the formula (length × width²)/2. After 5 weeks, the mice were euthanized by CO₂ asphyxiation, and the subcutaneous tumors were excised, photographed, and weighed. The collected tumor tissues were fixed in 4% paraformaldehyde for subsequent experiments. All animal experiments were strictly conducted in accordance with the ethical guidelines of Yangzhou University and were approved by the Animal Welfare and Ethics Committee of Yangzhou University (approval No. 202502911). In this study, the maximum tumor diameter did not exceed 1.5 cm, in compliance with the regulations of the Ethics Committee.

### Statistical analysis

All statistical analyses were performed using R software (version 4.3.3) and GraphPad Prism 10. The comparison of quantitative data between two groups was performed using the independent t-test, while multiple groups were compared using one-way analysis of variance (ANOVA) followed by post-hoc tests. Chi-square or Fisher’s exact tests were used for categorical variables. Kaplan–Meier survival analysis was conducted, and survival differences were compared using the log-rank test. A *p*-value < 0.05 was considered statistically significant.

## Results

### Bioinformatics analysis identifies FBXO5 overexpression in BC and Its prognostic significance

**Fig. 1:**
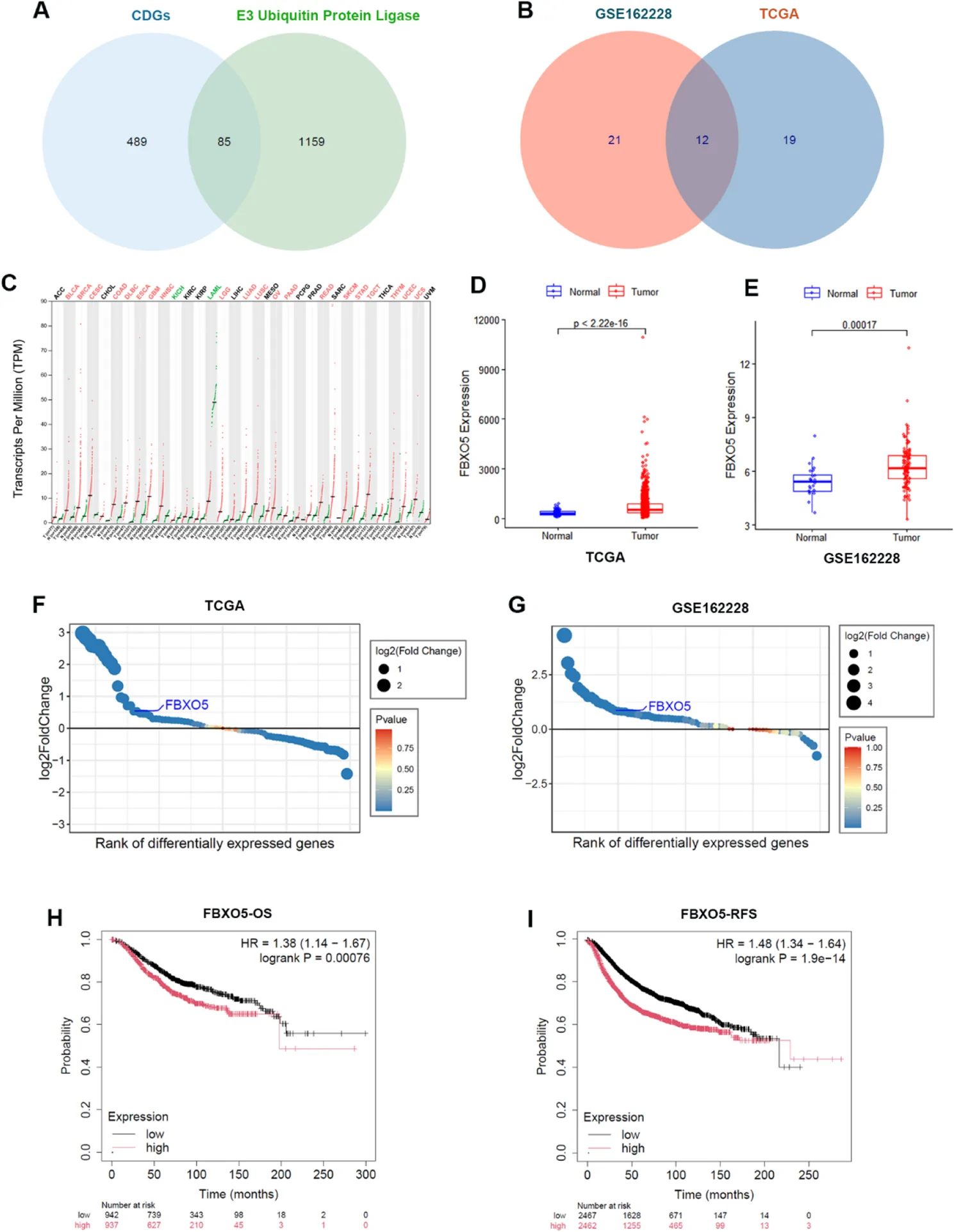
Bioinformatics analysis revealed FBXO5 overexpression in BC and Its poor prognosis. **A** Venn diagram showing the intersection of E3 ubiquitin ligase-related CDGs from the DepMap and UniProt databases, identifying 85 candidate genes. **B** Differential expression analysis of these 85 genes in the TCGA and GSE162228 datasets, leading to the selection of 12 potential oncogenes. **C** Pan-cancer expression analysis of FBXO5 (GEPIA). **D**,** E** Expression analysis of FBXO5 in BC using TCGA and GSE162228 datasets. **F**,** G** Volcano plots showing differential expression of E3 ubiquitin ligase-related CDGs in TCGA and GSE25488 datasets. **H-I** Kaplan-Meier survival curves showing the association between high FBXO5 expression and poorer OS and RFS in BC patients

To explore E3 ubiquitin ligase-related CDGs in BC we first screened 573 CDGs based on the DepMap database and intersected them with 1,244 E3 ubiquitin ligases from the UniProt database, identifying 85 E3 ubiquitin ligase-related CDGs(Fig. 1A). Subsequently, differential expression analysis of these 85 genes was performed using TCGA and GEO (GSE162228) databases, resulting in the identification of potential oncogenes associated with tumorigenesis. Ultimately, 12 E3 ubiquitin ligase-related CDGs were selected (Fig. 1B). Among them, FBXO5, a member of the F-box family, is involved in critical cellular biological functions such as the cell cycle and is highly expressed in various cancers, correlating with poor prognosis. However, its specific function and mechanisms in tumors have not been fully investigated. Therefore, we selected FBXO5 as the focus of this study to further explore its role and potential mechanisms in BC.

Pan-cancer analysis based on the GEPIA database demonstrated that FBXO5 was highly expressed in various cancer tissues compared to normal tissues (Fig. 1C). To verify the expression of FBXO5 in BC we further analyzed the TCGA and GEO (GSE162228) datasets. The results consistently showed that FBXO5 mRNA levels were significantly higher in BC tissues compared to adjacent normal tissues (Figs. 1D-E). Volcano plot analysis further indicated that FBXO5 is one of the significantly upregulated genes in both the TCGA and GSE162228 datasets (Figs. 1F-G).Additionally, survival analysis using the Kaplan-Meier Plotter database showed that high FBXO5 expression was significantly associated with poorer overall survival (OS) and relapse-free survival (RFS) in BC patients (Fig. 1H, I).

To explore the potential upstream mechanisms underlying FBXO5 overexpression in BC, we analyzed the genetic mutations and copy number variations of FBXO5 across the TCGA BC cohort via the cBioPortal database. Genomic alterations of FBXO5 were detected in only 1.71% of total cases, including amplification in 1.31% (13 samples), missense mutation in 0.2% (2 samples), and deep deletion in 0.2% (2 samples) (Supplementary Figure S1A).We further examined the DNA methylation status of FBXO5. UALCAN database analysis revealed no statistically significant difference in FBXO5 methylation levels between tumor and normal tissues (*p* = 0.126, Supplementary Figure S1B). Meanwhile, correlation analysis performed on cBioPortal demonstrated no significant association between FBXO5 methylation and its mRNA expression (Spearman *r* = 0.04, *p* = 0.259; Pearson *r* = 0.05, *p* = 0.0986, Supplementary Figure S1C).These results indicate that CNV and methylation are not major drivers of FBXO5 upregulation in BC.

### High expression of FBXO5 in BC cells and tissues is associated with poor prognosis

**Fig. 2:**
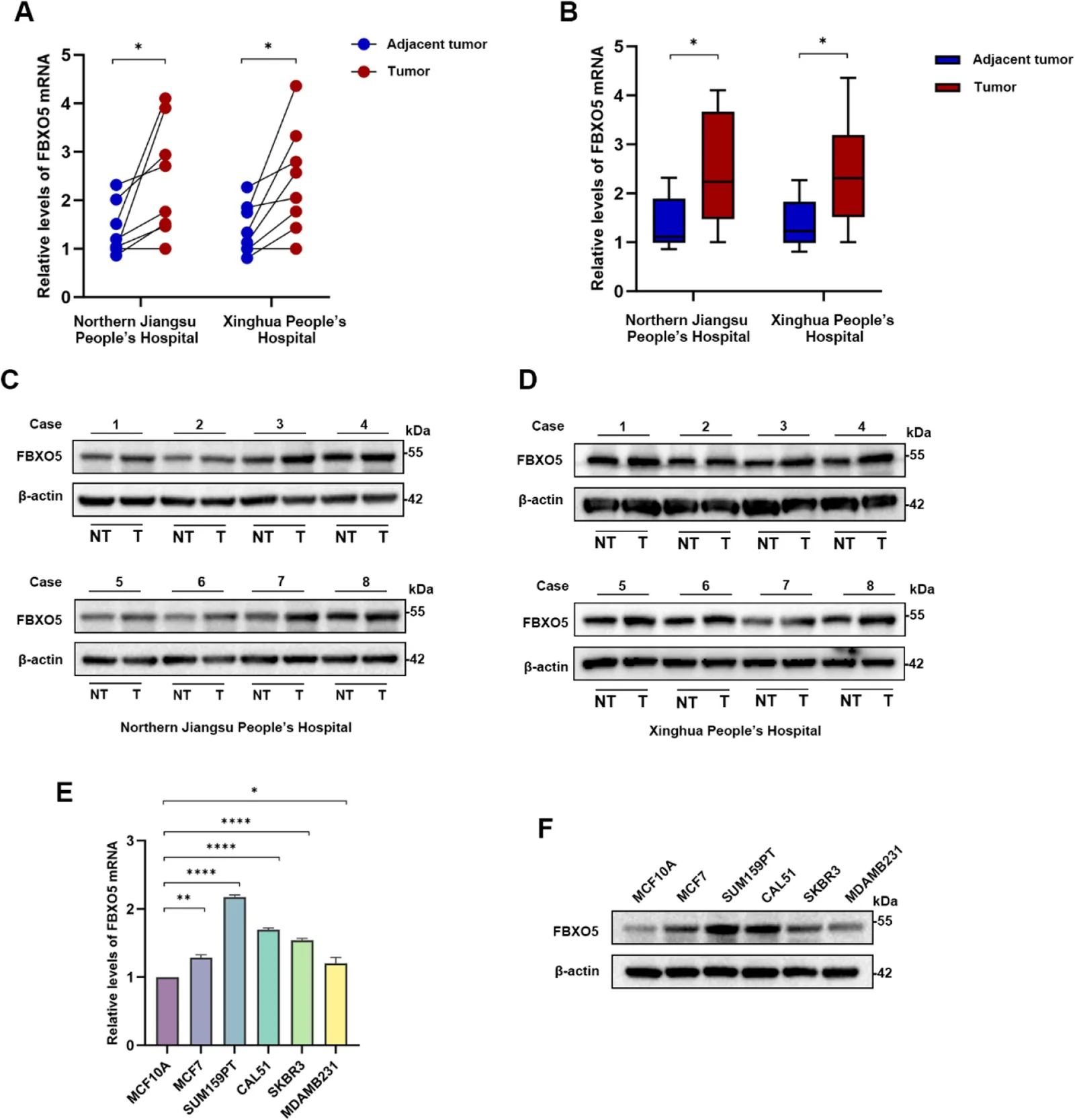
Expression of FBXO5 in BC tissues and cell lines. **A**,** B** FBXO5 mRNA expression in BC tissues and matched adjacent normal tissues: qRT-PCR analysis (**A**) overall comparison and (**B**) paired comparison. **C**,** D** WB detection of FBXO5 expression in BC and matched adjacent normal tissues from 8 pairs of samples collected from two centers. **E**,** F** qRT-PCR(E) and WB(F) to detect FBXO5 expression in normal breast epithelial cell line MCF10A and BC cell lines. *p < 0.05, **p < 0.01, ****P < 0.0001

Through bioinformatics analysis, we initially found that FBXO5 is highly expressed in BC tissues and is associated with poor prognosis. To further validate the expression of FBXO5 in BC, we performed qRT-PCR and Western blot analysis on 8 pairs of fresh BC and matched adjacent normal tissue samples from two centers. The results showed that, both in paired comparisons and at the overall level, FBXO5 mRNA expression was significantly higher in BC tissues compared to adjacent normal tissues (Fig. 2A, B). At the protein level, FBXO5 expression in BC tissues was also higher than in adjacent normal tissues (Fig. 2C, D). Additionally, we examined the expression of FBXO5 in normal breast epithelial cells (MCF10A) and various BC cell lines (MCF7, SUM159PT, CAL51, SKBR3, MDA-MB-231). Both qRT-PCR and Western blot results demonstrated that FBXO5 mRNA and protein expression were significantly elevated in BC cell lines compared to MCF10A cells (Fig. 2E-F).

**Fig. 3:**
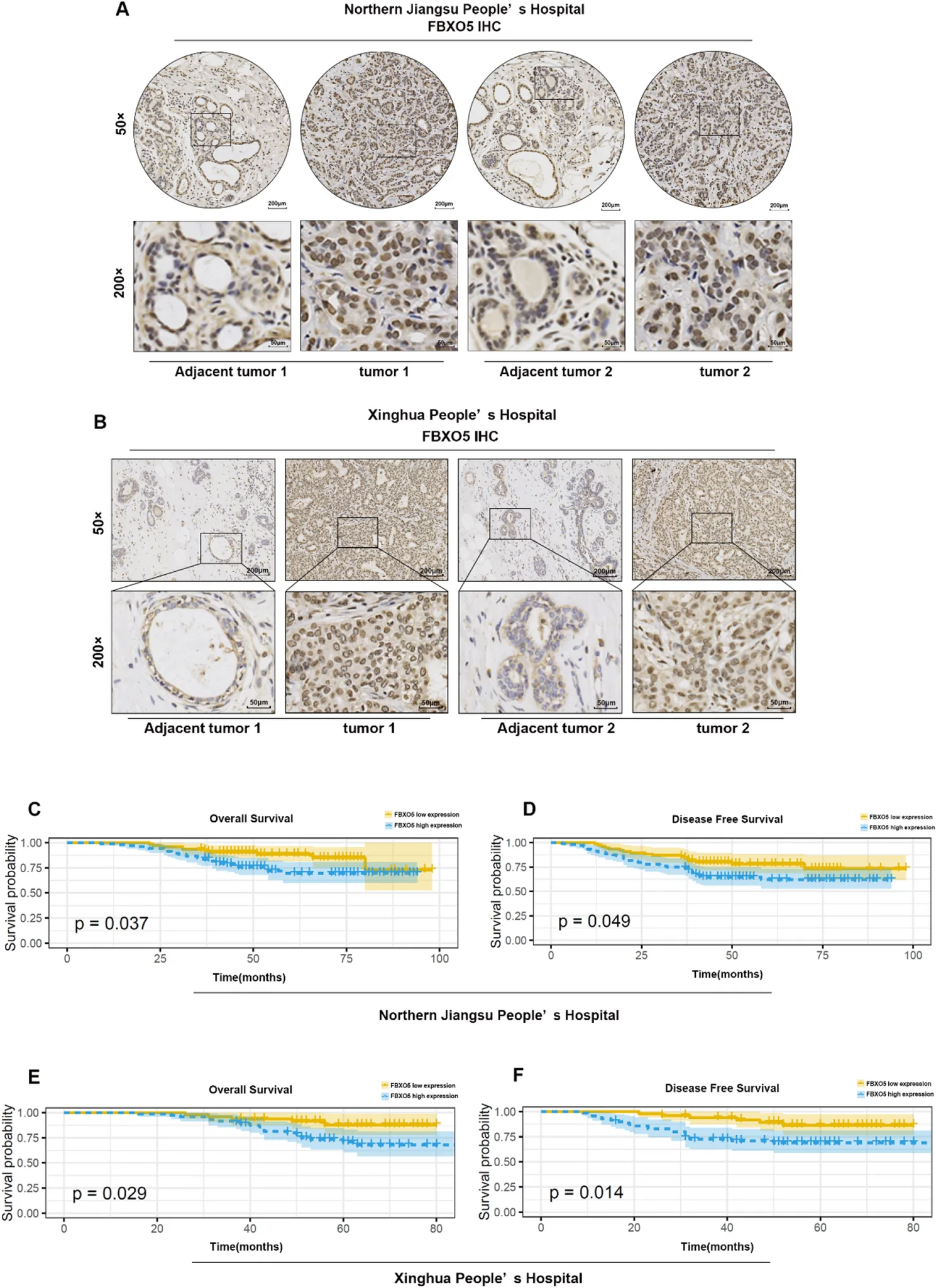
FBXO5 expression in BC tissues and its clinical significance. **A**,** B** IHC detection of FBXO5 expression in BC tissue microarrays (**A**) and conventional tissue sections (**B**), comparing cancer tissues with matched adjacent normal tissues. **C-F** High expression of FBXO5 is associated with poor OS and DFS in patient samples collected from two centers

We performed immunohistochemistry (IHC) analysis on BC tissue samples collected from two centers. The results consistently showed that, compared to adjacent normal tissues, the protein expression level of FBXO5 was significantly higher in BC tissues, as evidenced by stronger staining intensity, higher positivity rates, and corresponding higher staining scores (Fig. 3A, B). To further explore the clinical significance of FBXO5, we categorized patients into high and low FBXO5 expression groups based on IHC scores and analyzed its association with clinical and pathological features. The results indicated that in both cohorts, FBXO5 expression levels were significantly correlated with tumor size, histological grade, lymph node status, and high Ki67 index (*p* < 0.05), but showed no significant correlation with patient age, HER2 status, estrogen receptor (ER) status, or progesterone receptor (PR) status (Tables 1 and 2). Further Kaplan-Meier survival analysis demonstrated that FBXO5 overexpression was significantly associated with poorer overall survival (OS) and disease-free survival (DFS)( Fig. 3C-F), a trend that was confirmed in samples from both centers.

**Table 1:** Relationship between FBXO5 expression and clinicopathological characteristics of BC patients at Northern Jiangsu People's Hospital

Clinicopathological features		FBXO5 Expression Level		x2	P-value
		Low	High		
Total cases		76	104		
Age	>55	23 (30.3)	47 (45.2)	3.513887428	0.061
	≤55	53 (69.7)	57 (54.8)		
Tumor size	>2cm	42 (55.3)	89 (85.6)	18.86586853	<0.001
	≤2cm	34 (44.7)	15 (14.4)		
Grade	I-II	52 (68.4)	54 (51.9)	4.27883325	0.039
	III	24 (31.6)	50 (48.1)		
Lymph Node Status	No metastasis	45 (59.2)	39 (37.5)	7.466483968	0.006
	Metastasis	31 (40.8)	65 (62.5)		
ER	Negative	27 (35.5)	45 (43.3)	0.798013664	0.372
	Positive	49 (64.5)	59 (56.7)		
PR	Negative	36 (47.4)	55 (52.9)	0.336625004	0.562
	Positive	40 (52.6)	49 (47.1)		
HER2	Negative	54 (71.1)	71 (68.3)	0.055980861	0.813
	Positive	22 (28.9)	33 (31.7)		
Ki67	<30	49 (64.5)	25 (24.0)	28.00856711	<0.001
	≥30	27 (35.5)	79 (76.0)		

1

**Table 2:** Relationship between FBXO5 expression and clinicopathological characteristics of BC patients at Xinghua People's Hospital

Clinicopathological features		FBXO5 Expression Level		x2	*P*-value
		Low	High		
Total cases		48	72		
Age	>55	31 (64.6)	41 (56.9)	0.418113426	0.518
	≤55	17 (35.4)	31 (43.1)		
Tumor size	>2cm	18 (37.5)	48 (66.7)	8.755611672	0.003
	≤2cm	30 (62.5)	24 (33.3)		
Grade	I-II	31 (64.6)	30 (41.7)	5.169491525	0.023
	III	17 (35.4)	42 (58.3)		
Lymph Node Status	No metastasis	36 (92.3)	35 (71.4)	4.808077077	0.028
	Metastasis	3 (7.7)	14 (28.6)		
ER	Negative	15 (31.2)	28 (38.9)	0.436424041	0.509
	Positive	33 (68.8)	44 (61.1)		
PR	Negative	19 (39.6)	36 (50.0)	0.874125874	0.35
	Positive	29 (60.4)	36 (50.0)		
HER2	Negative	35 (72.9)	42 (58.3)	2.067351253	0.15
	Positive	13 (27.1)	30 (41.7)		
Ki67	<30	39 (81.2)	24 (33.3)	24.62962963	<0.001
	≥30	9 (18.8)	48 (66.7)		

### FBXO5 impact on BC cell behaviour in vitro and vivo

**Fig. 4:**
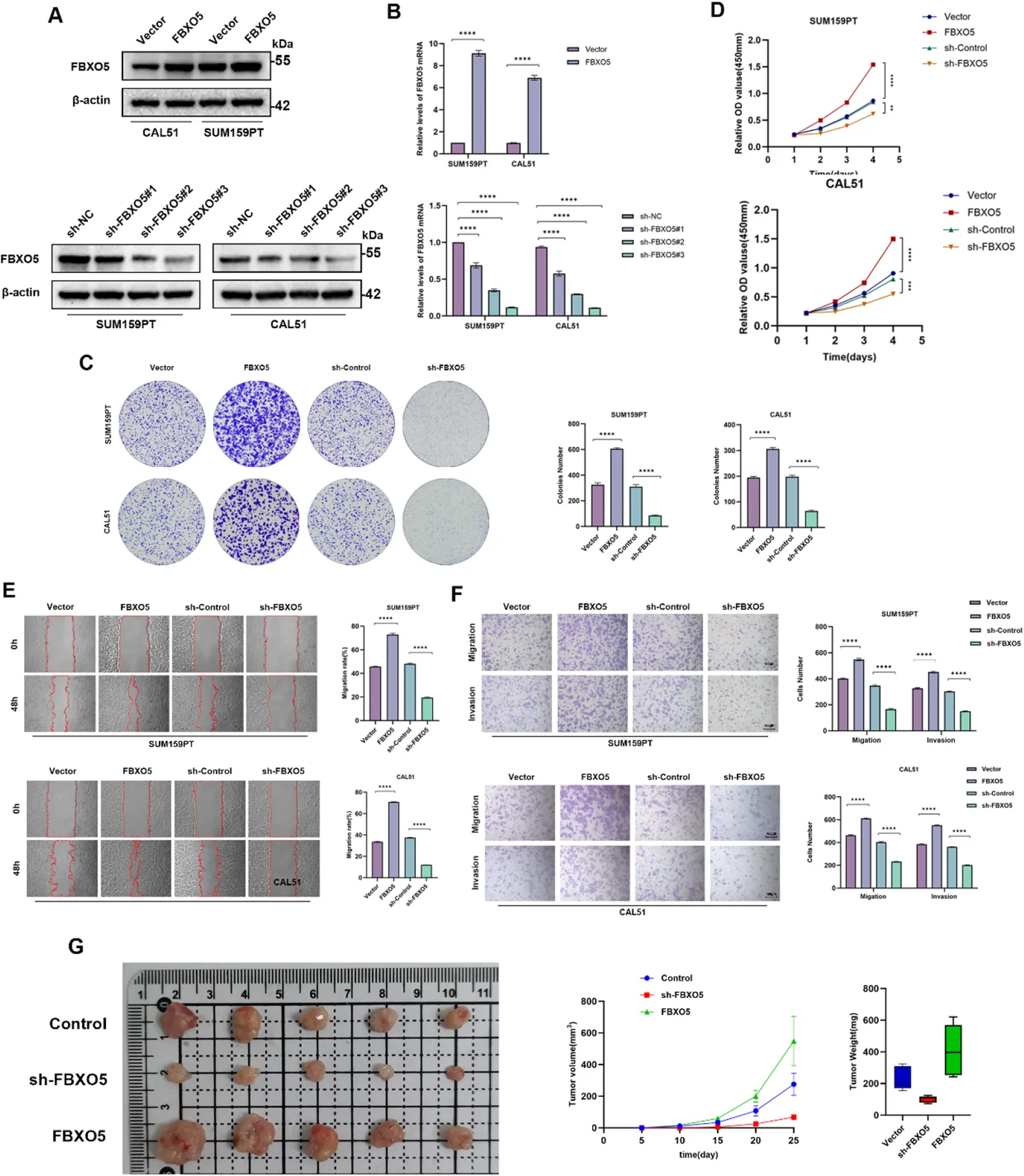
FBXO5 regulates the malignant biological behaviors of BC cells in vitro and in vivo. **A**,** B** qRT-PCR (**A**) and Western blot (**B**) assay for the construction of stably transfected overexpressing and knockdown FBXO5 BC cells. **C**,** D** Clone formation (**C**) and CCK-8 (**D**) assays to evaluate the effect of FBXO5 on the proliferative capacity of BC cells. **E** Wound healing assay was used to evaluate the effect of FBXO5 on cell migration. **F** Transwell assays were used to assess the effect of FBXO5 on cell migration and invasion. **G** Subcutaneous tumorigenesis experiment in nude mice to evaluate the tumor-promoting effect of FBXO5 in vivo. **p < 0.01, ***p < 0.001,****P < 0.0001

FBXO5 is highly expressed in BC tissues and cells and is significantly associated with poor prognosis in patients. Based on these findings, we hypothesize that FBXO5 may play an oncogenic role in BC. To test this hypothesis, we selected the SUM159PT cell line, which is highly dependent on FBXO5, and the CAL51 cell line, which has low FBXO5 dependency, as cell lines for further experiments based on the CERES score from the DepMap database. We constructed stable FBXO5 overexpression, knockdown, and empty vector control cell lines using lentiviral vectors, and the successful establishment of these cell models was confirmed by qRT-PCR and Western blot (Fig. 4A-B).

In vitro functional assays demonstrated that FBXO5 significantly promotes the malignant phenotype of BC cells. Colony formation assays indicated that overexpression of FBXO5 significantly enhanced the proliferative ability of BC cells, while knockdown of FBXO5 markedly inhibited their proliferation (Fig. 4C). CCK-8 assays further confirmed that FBXO5 overexpression significantly boosted cell viability, while its knockdown reduced it (Fig. 4D). In terms of cell migration and invasion, wound healing assays confirmed that FBXO5 overexpression significantly enhanced the migratory capacity of BC cells, while FBXO5 knockdown reduced their migration ability (Fig. 4E). Transwell assays also indicated that FBXO5 overexpression significantly promoted both migration and invasion of BC cells, while FBXO5 knockdown significantly inhibited these processes (Fig. 4F).

To further validate the tumor-promoting effect of FBXO5 in vivo, we performed subcutaneous tumorigenesis experiments in nude mice. The results showed that mice inoculated with SUM159PT BC cells overexpressing FBXO5 developed larger tumor volumes, heavier tumor weights, and faster growth rates, while those inoculated with FBXO5 knockdown cells exhibited smaller tumor volumes, lighter tumor weights, and slower growth rates (Fig. 4G).

Taken together, these in vitro and in vivo experimental results indicate that FBXO5 plays a crucial role in regulating the malignant biological behaviors of BC cells, including proliferation, migration, and invasion.

### FBXO5 Interaction with RPL23A

**Fig. 5:**
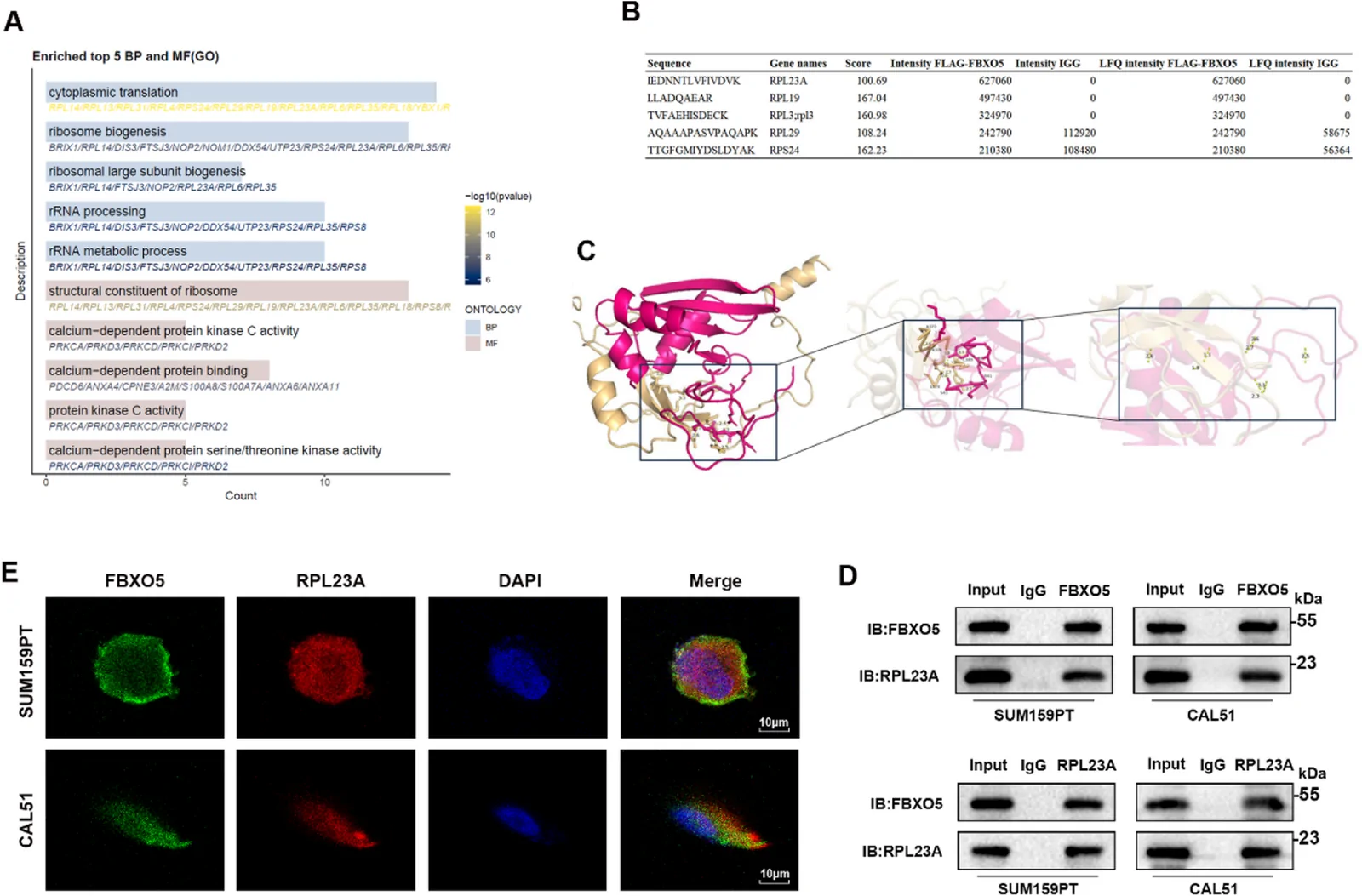
FBXO5 interaction with RPL23A. **A** Gene Ontology (GO) enrichment analysis of proteins interacting with FBXO5. **B** Mass spectrometry analysis showing the abundance of ribosomal proteins interacting with FBXO5, with RPL23A showing the highest enrichment. **C** Molecular docking analysis demonstrating stable interactions between FBXO5 and RPL23A through multiple hydrogen bonds. **D** CO-IP analysis showing an endogenous direct interaction between FBXO5 and RPL23A. **E** Immunofluorescence confocal microscopy results showing the cellular localization of FBXO5 and RPL23A

To investigate the potential target proteins of FBXO5 in BC, we first performed co-immunoprecipitation (CO-IP) in cells overexpressing FBXO5. Through CO-IP followed by mass spectrometry analysis, we identified 117 potential proteins that may interact with FBXO5. Gene Ontology (GO) enrichment analysis revealed that the biological processes (BP) and molecular functions (MF) most significantly enriched in these proteins were primarily associated with ribosomal protein family functions, especially in key biological processes such as ribosome biogenesis and cytoplasmic translation (Fig. 5A). Among them, 13 ribosomal proteins showed significant enrichment in the analysis, suggesting that these proteins may play important roles in FBXO5-related cellular processes and functions. Notably, RPL23A, a member of the ribosomal protein family, showed high enrichment in critical processes such as cytoplasmic translation and ribosome biogenesis. Furthermore, its abundance value in the mass spectrometry analysis was the highest among all ribosomal protein genes interacting with FBXO5 (Fig. 5B). Therefore, we selected RPL23A as a potential interacting gene of FBXO5 for further investigation.

To verify whether RPL23A is a direct interacting protein of FBXO5, we first performed molecular docking analysis. The docking results indicated that FBXO5 and RPL23A could form stable interactions through multiple hydrogen bonds, with significant spatial complementarity at the binding interface (Fig. 5C), suggesting the possibility of a direct structural interaction between the two. Next, co-immunoprecipitation (CO-IP) results showed an endogenous direct interaction between FBXO5 and RPL23A (Fig. 5D). Furthermore, to further investigate their cellular localization, immunofluorescence confocal microscopy results demonstrated that FBXO5 and RPL23A were primarily localized in both the cytoplasm and the nucleus (Fig. 5E).

### Regulation of RPL23A protein expression by FBXO5

**Fig. 6:**
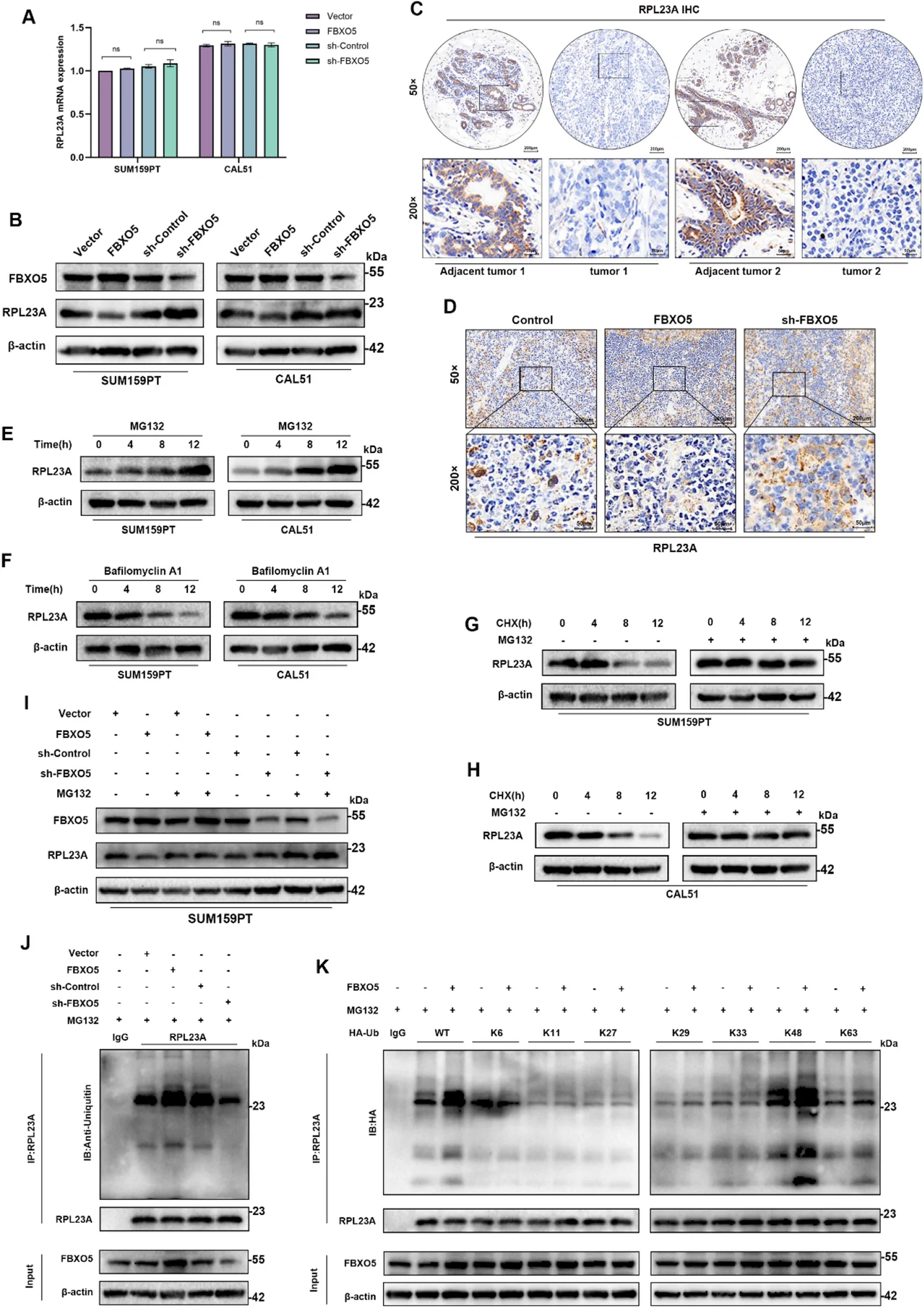
FBXO5 promotes RPL23A degradation and ubiquitination. **A**,** B** qRT-PCR (**A**) and WB (**B**) assay for the relationship between PELI1 and RPS3 expression in PC cells. **C** IHC analysis of tissue samples from Northern Jiangsu People’s Hospital showed low RPL23A expression in tumor tissues and high expression in adjacent non-tumor tissues. **D** IHC analysis of xenograft tumors showed a negative correlation between FBXO5 expression and RPL23A levels. **E**,** F** WB analysis revealed that RPL23A protein levels accumulated over time in response to MG132 treatment(**E**), while Bafilomycin A1 had no significant effect (**F**). **G**,** H** Treatment with CHX extended the half-life of RPL23A in the presence of MG132.**I** The degradation of RPL23A protein by FBXO5 was inhibited after MG132 treatment. **J** FBXO5 expression was positively correlated with RPL23A ubiquitination. **K** RPL23A exhibited the highest level of ubiquitination at the K48 chain, and FBXO5 overexpression enhanced ubiquitination at this site

To further investigate the regulatory mechanism between FBXO5 and RPL23A, we first measured the mRNA expression levels of RPL23A in SUM159PT and CAL51 cells after knockdown or overexpression of FBXO5 using qRT-PCR. The results showed no significant difference in the mRNA expression levels of RPL23A (Fig. 6A). Subsequently, we assessed the protein levels of RPL23A through Western blot analysis. The results indicated that upon overexpression of FBXO5, the protein expression level of RPL23A was significantly reduced, whereas knockdown of FBXO5 led to a significant increase in RPL23A protein levels (Fig. 6B). We performed IHC on RPL23A using tissue microarrays from Northern Jiangsu People’s Hospital. The results showed that the expression level of RPL23A in tumor was significantly lower than that in adjacent non-tumor tissues(Figure 6C). This expression difference may be closely associated with the elevated expression of FBXO5 in tumer, suggesting that FBXO5 may negatively regulate RPL23A. Furthermore, IHC analysis of xenograft tumors in nude mice also indicated that modulation of FBXO5 affects RPL23A protein expression: overexpression of FBXO5 led to a decrease in RPL23A levels, while FBXO5 knockdown resulted in an increase in RPL23A expression(Fig. 6D).Therefore, although the mRNA transcription levels of RPL23A did not show significant changes after FBXO5 knockdown or overexpression, its translation level exhibited noticeable changes. This suggests that the expression of RPL23A may be regulated by FBXO5 through post-translational modifications.

To elucidate the mechanism of FBXO5-mediated post-translational modifications of RPL23A, we first investigated its degradation pathway through protein stability assays. After treating cells with the proteasome inhibitor MG132 and the lysosome inhibitor Bafilomycin A1, Western blot results showed that RPL23A protein levels accumulated in a time-dependent manner following MG132 treatment, while no significant effect was observed with Bafilomycin A1 treatment (Fig. 6E, F), suggesting that RPL23A is primarily degraded through the proteasomal pathway. Subsequently, in the presence of MG132, we co-treated cells with the protein synthesis inhibitor CHX and observed a significant extension in the half-life of RPL23A (Fig. 6G, H), confirming that its degradation is dynamic and proteasome-dependent. To directly confirm the regulatory role of FBXO5, we applied MG132 in FBXO5-overexpressing and knockdown cells. The results showed that the reduction in RPL23A protein levels induced by FBXO5 overexpression could be reversed by MG132, whereas no significant difference in RPL23A protein levels was observed in FBXO5 knockdown cells compared to the control group, indicating that FBXO5 is a key regulatory factor in the ubiquitin-proteasome-mediated degradation of RPL23A (Fig. 6I).

To further elucidate the mechanism, we directly measured the ubiquitination levels of RPL23A. The results showed that overexpression of FBXO5 increased the ubiquitination levels of RPL23A, whereas knockdown of FBXO5 led to a decrease in RPL23A ubiquitination levels (Fig. 6J). To further investigate the key ubiquitination site of RPL23A regulated by FBXO5, we constructed HA-tagged ubiquitin chain plasmids and co-transfected them with RPL23A plasmids into control and FBXO5-overexpressing cells. Western blot analysis revealed that RPL23A exhibited the highest level of ubiquitination at the K48 chain, and FBXO5 overexpression promoted RPL23A ubiquitination at the K48 chain (Fig. 6K).

### FBXO5 modulates MDM2-p53 pathway via RPL23A

**Fig. 7:**
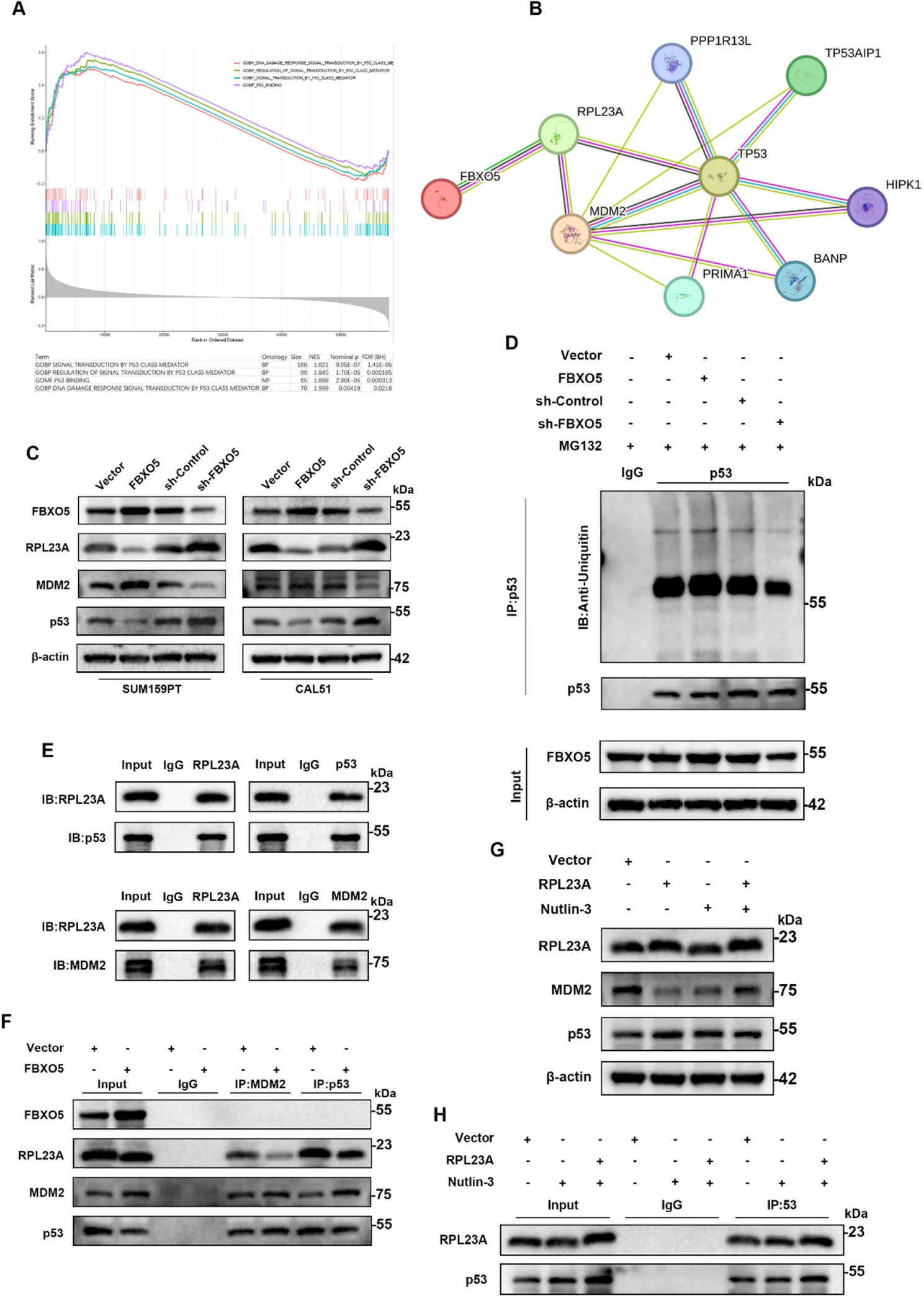
FBXO5 regulates the MDM2-p53 axis through RPL23A-mediated ubiquitination. **A** GSEA analysis of TCGA data showed significant enrichment of p53-related functions associated with FBXO5. **B** PPI network analysis identified potential upstream and downstream regulatory relationships between FBXO5, RPL23A, MDM2, and p53. **C**,** D** WB analysis showed that FBXO5 expression promoted p53 degradation and ubiquitination. **E**,** F** CO-IP assays confirmed a direct interaction between RPL23A, MDM2, and p53(**E**), with FBXO5 overexpression weakening the RPL23A-MDM2 interaction and enhancing the MDM2-p53 interaction(**F**). **G**,** H** Treatment with the MDM2 inhibitor Nutlin-3 showed that overexpression of RPL23A enhanced its interaction with p53(**H**), but did not significantly affect p53 protein levels(**G**)

To further elucidate the oncogenic mechanism by which FBXO5 promotes BC through the regulation of RPL23A, we performed Gene Set Enrichment Analysis (GSEA) using data from the TCGA database to identify genes associated with FBXO5. The results showed significant enrichment in several functional pathways, including p53-mediated signaling, regulation, and p53 binding activity (Fig. 7A). It is known that the stability of p53 is primarily regulated by ubiquitination mediated by MDM2, affecting various cellular processes and pathways [23–25]. Additionally, the ribosomal protein (RPs)-MDM2-p53 pathway, which has recently been identified as influencing p53 protein activity, involves several ribosomal proteins in the regulation of p53 [26–33]. Based on the results from our study, we hypothesize that FBXO5 may regulate MDM2-p53 signaling by mediating RPL23A. To validate this hypothesis, we first performed protein-protein interaction (PPI) network analysis, which revealed potential upstream and downstream regulatory relationships between FBXO5, RPL23A, MDM2, and p53 (Fig. 7B). Subsequently, Western blot results showed that overexpression of FBXO5 in BC cells led to a decrease in p53 protein levels, while knockdown of FBXO5 resulted in increased p53 expression (Fig. 7C). Further ubiquitination assays revealed a negative correlation between FBXO5 expression and p53 ubiquitination levels (Fig. 7D), suggesting that FBXO5 may regulate p53 stability by affecting its ubiquitination modification.

To validate whether FBXO5 regulates the MDM2-p53 interaction through RPL23A, we first confirmed the direct interaction between RPL23A, MDM2, and p53 through CO-IP assays (Fig. 7E). Further multi-protein CO-IP results showed that FBXO5 does not directly interact with MDM2 or p53. However, overexpression of FBXO5 weakened the interaction between RPL23A and MDM2 while enhancing the interaction between MDM2 and p53 (Fig. 7F). These results suggest that FBXO5 may indirectly regulate MDM2-mediated p53 ubiquitination through the mediation of RPL23A.

To investigate whether the interaction between RPL23A and p53 directly affects p53 stability, we performed verification under conditions treated with the MDM2 inhibitor (Nutlin-3). The results showed that although overexpression of RPL23A enhanced its interaction with p53, there was no significant change in p53 protein levels. These results suggest that the regulation of p53 by RPL23A is dependent on MDM2, rather than through direct modulation of p53 stability (Fig. 7G, H).

### FBXO5 promotes malignant behaviors of BC by regulating the RPL23A/MDM2/p53 axis

**Fig. 8:**
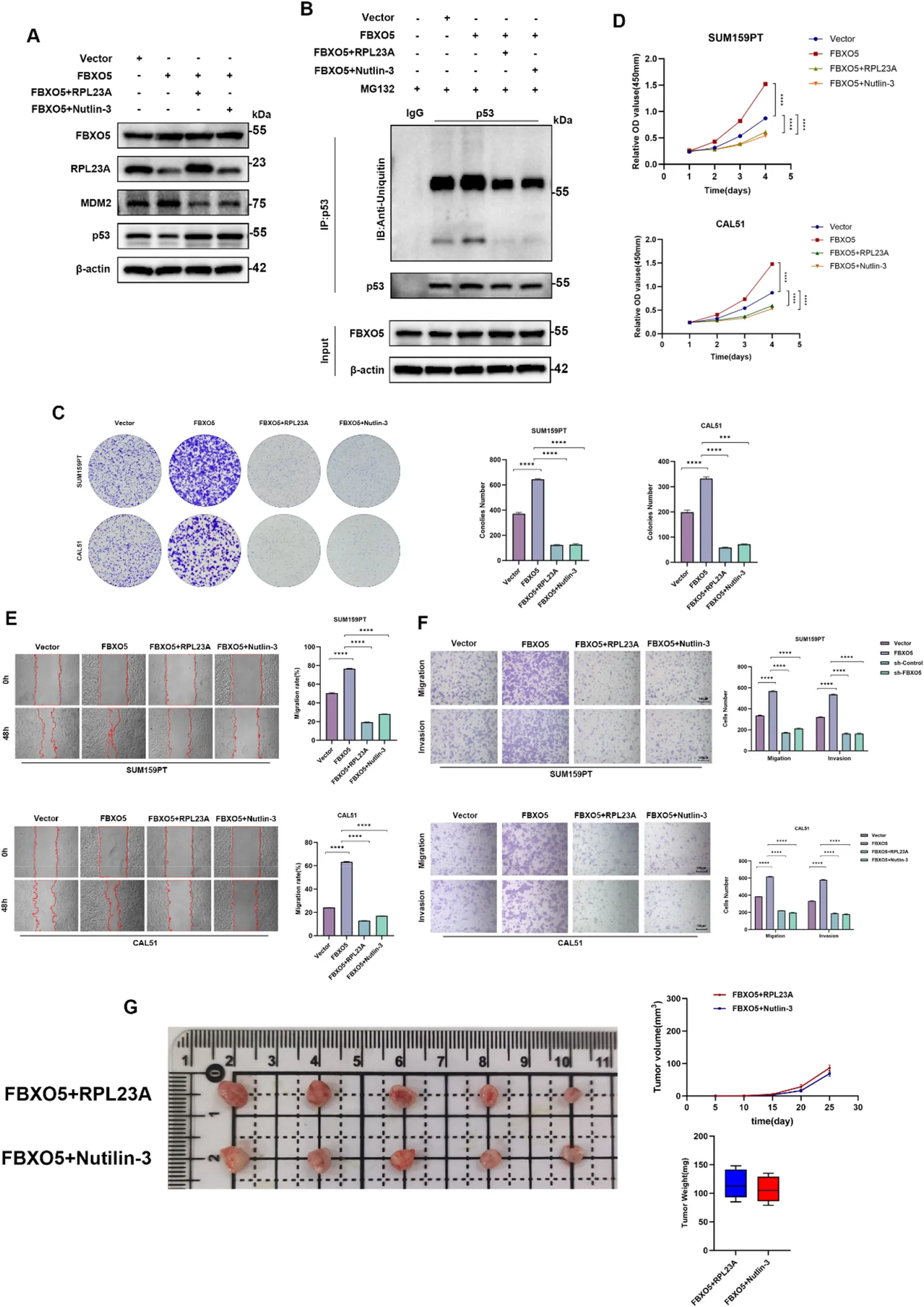
FBXO5 promotes malignant behaviors of BC through the RPL23A/MDM2/p53 axis. **A**,** B** WB and ubiquitination assays showed that RPL23A overexpression or Nutlin-3 treatment reversed the effect of FBXO5 overexpression on p53 protein levels and ubiquitination. **C**,** D** Colony formation and CCK-8 assays showed that RPL23A overexpression or Nutlin-3 treatment effectively reversed the enhanced proliferation induced by FBXO5 overexpression. **E**,** F** Wound healing assays showed that RPL23A overexpression or Nutlin-3 treatment suppressed the FBXO5-induced migration of BC cells(**E**); whereas Transwell assays confirmed that these treatments reduced both the migration and invasion of BC cells enhanced by FBXO5(**F**). **G** Subcutaneous tumorigenesis in nude mice showed that RPL23A overexpression or Nutlin-3 treatment reversed the tumor-promoting effect of FBXO5 overexpression. ****P < 0.0001

To further validate the oncogenic mechanism of FBXO5 through the regulation of the RPL23A/MDM2/p53 axis in BC, we conducted rescue experiments. The experimental groups included: control group, FBXO5 overexpression group, combined FBXO5 and RPL23A overexpression group, and FBXO5 overexpression group treated with the MDM2 inhibitor Nutlin-3. Western blot analysis revealed that both co-expression of RPL23A and treatment with Nutlin-3 reversed the decrease in p53 protein levels induced by FBXO5 overexpression, indicating that FBXO5’s regulation of p53 is dependent on RPL23A and mediated through MDM2 (Fig. 8A). Further ubiquitination assays supported this conclusion, as RPL23A overexpression or Nutlin-3 treatment effectively antagonized the promoting effect of FBXO5 on p53 ubiquitination (Fig. 8B).

To further validate the biological impact of this pathway at the functional level, we conducted in vitro rescue experiments. Colony formation assay results showed that overexpression of RPL23A or treatment with Nutlin-3 effectively inhibited the enhanced proliferative ability of BC cells induced by FBXO5 overexpression (Fig. 8C). CCK-8 assays further confirmed that both RPL23A overexpression and Nutlin-3 treatment significantly reversed the increased cell viability caused by FBXO5 overexpression (Fig. 8D). Wound healing assay results demonstrated that both RPL23A overexpression and Nutlin-3 intervention effectively suppressed the increased migratory ability of cells induced by FBXO5 overexpression (Fig. 8E). Additionally, Transwell assays showed that both RPL23A overexpression and Nutlin-3 treatment significantly diminished the promotive effects of FBXO5 overexpression on BC cell migration and invasion (Fig. 8F).

The subcutaneous tumorigenesis experiment in nude mice showed a consistent trend. For effective comparison, we compared the newly added experimental groups (FBXO5/RPL23A co-expression group and FBXO5 overexpression + Nutlin-3 group) with the FBXO5 overexpression and control groups in this study. The results showed that tumor growth rate, final volume, and weight were significantly reduced in both intervention groups (Fig. 8G).

In summary, both molecular and functional in vitro and in vivo rescue experiments collectively demonstrate that FBXO5 promotes the ubiquitin-mediated degradation of RPL23A, thereby attenuating its inhibitory effect on MDM2, enhancing MDM2-mediated p53 degradation, and ultimately promoting the malignant progression of breast cancer.

## Discussion

Despite significant advancements in the study of the ubiquitin-proteasome system in various cancers [11, 34–36], the specific mechanisms of many ubiquitin enzymes in tumor progression remain unclear [37]. Therefore, exploring the detailed mechanisms of the ubiquitin-proteasome system in BC not only helps deepen our understanding of the disease’s etiology but also may provide potential directions for the development of new therapeutic targets.

**Fig. 9:**
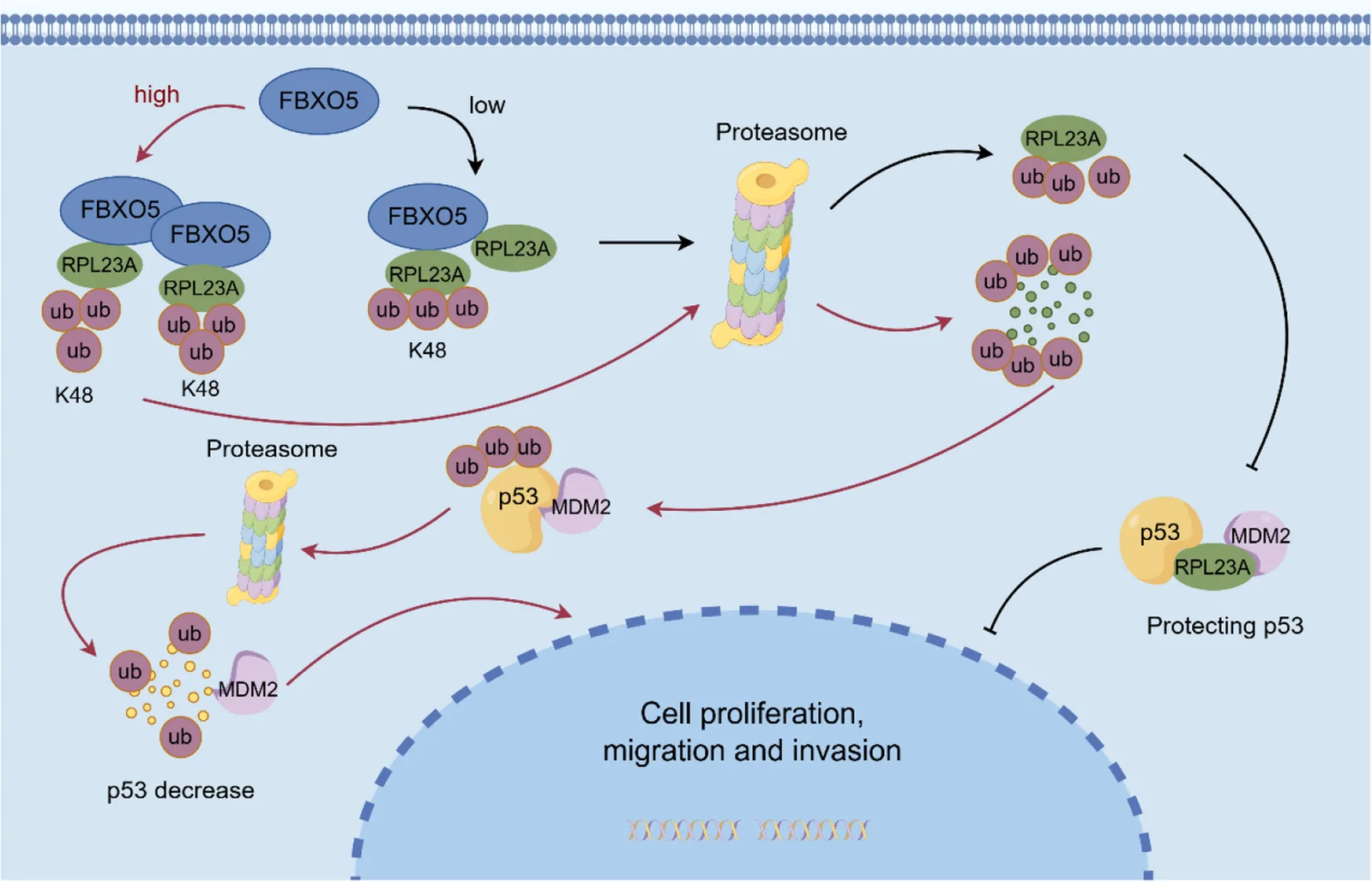
Mechanistic map of FBXO5 regulating the RPL23A/MDM2/p53 signaling pathway. FBXO5 promotes the ubiquitin-mediated degradation of RPL23A through the K48 ubiquitin site, which subsequently enhances MDM2-mediated degradation of p53, ultimately driving the malignant biological behaviors of breast cancer cells. Created by Figdraw.( Agreement number: YSSWUcdbed)

This study utilized the DepMap database, constructed using CRISPR-based methods, to systematically identify FBXO5, an E3 ubiquitin ligase, as a critical factor for the survival of BC cells. Existing studies have shown that FBXO5, as a specific component of the ubiquitin ligase complex, can mediate the ubiquitination of target proteins, thereby regulating their degradation and related biological processes [38–40]. Combining bioinformatics analysis, clinical sample validation, and in vitro and in vivo experiments, this study thoroughly investigates the oncogenic role and molecular mechanisms of FBXO5 in BC. Our findings indicate that FBXO5 is abnormally overexpressed in BC, and its expression level is closely associated with malignant tumor progression and poor patient prognosis. FBXO5 promotes BC cell proliferation, migration, and invasion by mediating the ubiquitin-mediated degradation of RPL23A, thereby regulating the MDM2/p53 signaling pathway (Fig. 9).

We selected E3 ubiquitin ligase-related CDGs from the DepMap and Uniprot databases, and further identified FBXO5 through differential expression analysis using data from TCGA and GEO databases. Subsequently, the specificity of FBXO5 high expression in BC was confirmed at both the mRNA and protein levels in BC-related cell lines and clinical samples from two centers. Clinical correlation analysis revealed that FBXO5 expression levels were significantly associated with tumor size, histological grade, lymph node status, and Ki67 index, further supporting its close relationship with the malignant phenotype of BC. Survival analysis confirmed the potential of FBXO5 as a prognostic marker for poor outcomes in BC.

Functional validation experiments both in vitro and in vivo confirmed the oncogenic role of FBXO5. By constructing FBXO5 overexpression and knockdown cell models, we found that FBXO5 overexpression significantly enhanced the proliferation, migration, and invasion abilities of BC cells, and promoted tumor growth in a xenograft mouse model. In contrast, knockdown of FBXO5 attenuated these malignant phenotypes. These results indicate that FBXO5 is a key driver of malignant behavior in BC.

The core contribution of this study lies in elucidating the molecular mechanism of FBXO5. Through CO-IP, mass spectrometry analysis, immunofluorescence confocal microscopy, molecular docking, enrichment analysis, and subsequent validation, we identified RPL23A as a direct interacting partner of FBXO5 for the first time. Our study shows that FBXO5 promotes the degradation of RPL23A via the proteasomal pathway through polyubiquitination at the K48 linkage. Additionally, based on TCGA data, we performed GSEA enrichment analysis of FBXO5-related genes, suggesting that FBXO5 may be involved in the regulation of p53. Since the ubiquitination of p53 is mainly regulated by MDM2, which affects various cellular processes and pathways [23–25], the ribosomal proteins (RPs)-MDM2-p53 axis has recently been identified as a pathway that regulates p53 activity [26–28], with several ribosomal proteins shown to modulate p53 through this pathway [29–33]. However, the specific mechanism and functional role of RPL23A in the MDM2-p53 regulation pathway remain unclear. Therefore, we further investigated the mechanism by which FBXO5 regulates the MDM2/p53 pathway through mediating RPL23A ubiquitination. Experimental results demonstrate that FBXO5 degrades RPL23A, which weakens the binding between RPL23A and MDM2, thereby enhancing the interaction between MDM2 and p53 and promoting the ubiquitination and degradation of p53 by MDM2. Thus, this study provides the first clear evidence of the specific mechanism and functional role of RPL23A, a ribosomal protein, in BC through PRs-MDM2-p53 regulatory axis.

Furthermore, rescue experiments further validated this mechanism. Both exogenous overexpression of RPL23A and treatment with the MDM2 inhibitor Nutlin-3 effectively reversed the downregulation of p53 and the enhanced malignant phenotype of BC cells induced by FBXO5 overexpression. Therefore, the oncogenic effect of FBXO5 is primarily mediated through the RPL23A-MDM2-p53 pathway.

Our findings position the FBXO5/RPL23A node within the well-established p53 tumor suppressor network, revealing actionable therapeutic vulnerabilities. First, FBXO5 itself is a compelling drug target; disrupting its interaction with RPL23A could restore p53 function. Second, high FBXO5 expression may predict sensitivity to MDM2 inhibitors (e.g., Nutlin-3), as demonstrated in our rescue experiments, thus positioning it as a potential predictive biomarker for patient stratification in p53-pathway-targeted therapies. Consequently, this axis provides a rationale for combination strategies co-targeting FBXO5 and MDM2 to achieve synergistic p53 activation.

However, this study also has some limitations. First, although we validated that RPL23A interacts with MDM2 and confirmed that FBXO5-mediated RPL23A degradation impairs their binding to relieve MDM2 suppression and facilitate p53 degradation, the specific interaction domains between RPL23A and MDM2, as well as the precise molecular pattern by which RPL23A inhibits MDM2 activity, were not characterized in this study.Future studies will adopt truncated protein mutation and domain interaction verification assays to identify the core functional regions responsible for RPL23A-MDM2 binding, thereby further clarifying the refined mechanism of RPL23A-mediated MDM2 regulation and improving the integrity of the FBXO5-RPL23A-MDM2-p53 signaling network.

Second, we further explored the potential upstream regulatory mechanism of FBXO5 overexpression in BC by analyzing genomic alteration and DNA methylation profiles based on TCGA cohort data. The results demonstrated that the incidence of FBXO5 genomic mutation and copy number variation was extremely low, and no significant difference in FBXO5 methylation was observed between tumor and normal tissues. Moreover, methylation level was not significantly correlated with FBXO5 mRNA expression. These findings exclude CNV and methylation aberration as the major drivers of FBXO5 upregulation, indicating that its upstream regulatory mechanism remains to be fully elucidated.Subsequent studies will focus on screening and validating key upstream transcription factors that modulate FBXO5 transcriptional activity to uncover its dysregulated transcriptional network. In addition, the post-translational modification and protein stability regulation of FBXO5 were not investigated in the present study. Further exploration of FBXO5 ubiquitination and phosphorylation will help clarify its protein homeostasis regulation and comprehensively enrich the upstream regulatory mechanism and oncogenic function of FBXO5 in BC.

Third, although the in vitro and in vivo findings are significant, further studies are needed before clinical translation, including the exploration of the tumor microenvironment modulation effect of FBXO5. These unresolved issues provide important directions for future research.

## Conclusion

This study systematically elucidates the oncogenic role of FBXO5 in BC and, for the first time, proposes a novel mechanism by which FBXO5 regulates the MDM2/p53 signaling pathway through mediating the ubiquitination and degradation of RPL23A. Our research provides new insights into the role of the ubiquitin-proteasome system in BC and highlights the potential of FBXO5 as a prognostic biomarker and therapeutic target for BC. This finding not only deepens our understanding of the molecular mechanisms underlying BC but also lays the theoretical foundation for future clinical research and the development of therapeutic strategies.

## Supplementary Information

Supplementary Material 1.

Supplementary Material 2.

Supplementary Material 3.

## Data Availability

The data that support the findings of this study are available from the corresponding author upon reasonable request.

## Abbrevlations

BC: Breast cancer
FBXO5: F-Box Protein 5
RPL23A: Ribosomal protein L23a
CDGs: Cancer Dependency Genes
DepMap: The Cancer Dependency Map
UPS: Ubiquitin-Proteasome System
TCGA: The Cancer Genome Atlas
GEO: Gene Expression Omnibus
OS: Overall Survival
RFS: Recurrence-free survival
DFS: Disease-free survival
WB: Western blotting
IHC: Immunohistochemistry
CHX: Cycloheximide
Co-IP: Co-Immunoprecipitation
