# Supplementary material for: FBXO5 regulates RPL23A to promote MDM2-mediated p53 degradation and facilitate malignant progression of breast cancer
Source: Breast Cancer Res. 2026 Jun 25;28:152. doi: 10.1186/s13058-026-02335-3 (PMC13555960; doi:10.1186/s13058-026-02335-3)
Supplement: Supplementary file 1 [file 13058_2026_2335_MOESM3_ESM.pdf]

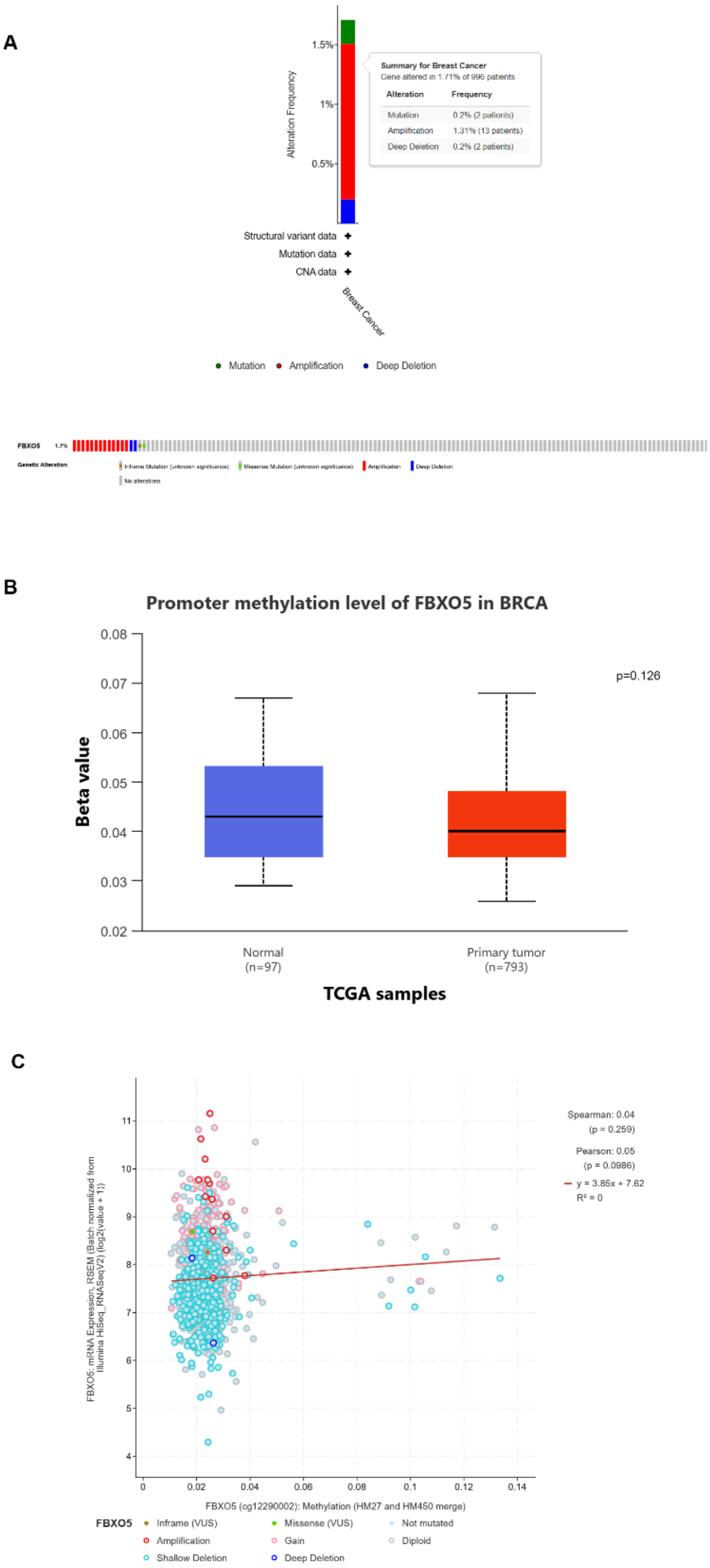


**Fig. S1** FBXO5 genomic alterations and methylation status in TCGA BC cohort. **A** Frequencies of FBXO5 genomic alterations, including amplification, missense mutation, and deep deletion, in 996 BC samples, with a total alteration frequency of 1.71%. **B** Comparison of FBXO5 methylation levels between breast cancer tissues and normal tissues ($p$ = 0.126). **C** Scatter plot showing correlation between FBXO5 methylation and mRNA expression (Spearman r = 0.04, $p$ = 0.259; Pearson r = 0.05, $p$ = 0.0986).
